# Role of Trifarotene in the Management of Acne in Indian Patients: Insights From an Indian Dermatology Experts' Meeting

**DOI:** 10.7759/cureus.65800

**Published:** 2024-07-30

**Authors:** D A Satish, Sanjeev Aurangabadkar, Sushil T Tahiliani, Rajetha Damisetty, Anurag Tiwari, Krupashankar D S, Nina Madnani, Abir Saraswat, Anupam Das, Dyotona Sen, Sameer Jadhwar

**Affiliations:** 1 Dermatology, Sagar Hospitals, Bengaluru, IND; 2 Dermatology, Aurangabadkar’s Skin & Laser Clinics, Hyderabad, IND; 3 Dermatology, Hinduja Hospital, Mumbai, IND; 4 Dermatology, Dr. Rajetha's Mohana Skin & Hair Clinic, Hyderabad, IND; 5 Dermatology, Anurag Tiwari Skin And Laser Clinic, Bhopal, IND; 6 Dermatology, Mallige Hospital, Bengaluru, IND; 7 Dermatology, Hinduja Clinic, Mumbai, IND; 8 Dermatology, Indushree Skin Clinic, Lucknow, IND; 9 Dermatology, KPC (Kali Pradip Chaudhuri) Medical College and Hospital, Kolkata, IND; 10 Medical Affairs, Galderma India Pvt Ltd, Mumbai, IND

**Keywords:** truncal acne, trifarotene, retinoids, disease management, acne vulgaris

## Abstract

Acne vulgaris, a prevalent inflammatory skin condition, significantly impacts individuals worldwide, particularly adolescents and adults. Its chronic nature, associated sequelae, and psychosocial impact underscore the substantial burden it poses. Current treatment guidelines primarily address facial acne, with limited guidance on managing truncal acne. Personalized approaches are increasingly recognized as essential for tailoring treatments to individual patient needs. This review integrates insights from an Indian Dermatology Experts' Meeting, featuring perspectives from nine leading dermatologists. Discussions centered on analyzing acne's burden, its effects on quality of life (QoL), unmet needs in management, trifarotene's role in Indian therapy, anticipated challenges, and the importance of ancillary care. The experts highlighted acne's profound impact on patients' QoL and identified gaps in current management guidelines, especially concerning truncal acne. Trifarotene, a fourth-generation topical retinoid approved by the FDA and Drug Controller General of India (DCGI) for facial and truncal acne, demonstrated safety and efficacy across age groups. This synthesis of expert perspectives underscores the need for personalized acne management. Trifarotene emerges as a promising therapeutic option but challenges remain, particularly in optimizing ancillary care to minimize treatment-related adverse effects. Addressing these issues will enhance treatment outcomes and patient satisfaction in acne management, emphasizing the importance of tailored approaches in clinical practice.

## Introduction and background

Acne vulgaris, also termed acne, is a common chronic inflammatory disorder of the pilosebaceous unit comprising sebaceous glands and hair follicles [1,2]. Globally, the condition, which occupies the eighth position among skin diseases, affects less than 9% of the population (irrespective of age) with the prevalence ranging from 35% to 100% in adolescents [3]. The condition is responsible for 4.96 million disability‑adjusted life years (DALY) and years lived with disability (YLD). In 2019, the Global Burden of Disease reported an upward trend in both DALY and YLD by 8% from 2010 to 2019 with no cases of mortality and years of life lost (YLL) due to acne [4]. Sebum hypersecretion, abnormal differentiation and proliferation of keratinocytes present in hair follicles, colonization of bacteria, and inflammatory host response are the four contributing factors to the development of acne [5]. However, the recently updated etiopathogenesis emphasizes inflammation as a key component for acne development. Increased activity of pro-inflammatory factors like interleukin-1 and cytokines begins before the initiation of hyperproliferation and is responsible for triggering the proliferation of keratinocytes. Additionally, other factors such as diet, the action of androgens and toll-like receptor-2 on keratinocytes, chemokines, neuroendocrine regulatory mechanism, recognition of molecular patterns associated with pathogens, and immune response also play a multifactorial role in the pathogenesis of acne [6].

Clinically, the condition is characterized by the presence of open or closed comedones (non‑inflammatory lesions) and nodules, papules, and pustules (inflammatory lesions) [7], occurring on the centrofacial area, upper trunk, back, and deltoid regions [1]. Although acne is considered a self-limiting condition that initiates at puberty and resolves gradually by early adulthood or late adolescence, few may experience a chronic course and continue to have acne until their late 50s [8-10]. The condition is concerning as it negatively impacts patients’ quality of life (QoL). Acne sequelae include post-inflammatory hyperpigmentation (PIH), common in skin phototypes IV-VI [2], and scarring (boxcar scars, ice pick scars, and rolling scars). These sequelae have a psychological impact, such as poor self-esteem, feelings of anger, shame, stigmatization, and concern, disturbed professional and personal relationships, social aversion, and, sometimes, limiting choice of clothing as well. The psychiatric impact of acne comprises anxiety, depression, and even suicidal thoughts [11].

The current management options for acne include skin hygiene, topical therapy, and systemic therapy. Topical therapy for acne mainly includes the use of first- and third-generation retinoids like tretinoin, adapalene, and tazarotene. Based on the severity of acne, these retinoids are usually combined with benzoyl peroxide (BPO) or antibiotics (topical or systemic). Systemic therapy comprises the oral use of retinoids (isotretinoin), antibiotics (minocycline, doxycycline, and sarecycline), and hormone therapy (oral contraceptives, spironolactone, and corticosteroids) [2]. Despite being effective, these treatments have adverse effects such as erythema, stinging sensations, skin dryness and irritation, retinoid flares due to the use of retinoids, and the development of antibiotic resistance due to the use of oral and topical antibiotics [12].

Around 30-60% of patients with facial acne also have truncal acne, although the condition often remains underreported as patients do not adequately report their truncal acne, and it is commonly underdiagnosed and undertreated by clinicians [13,14]. Additionally, the current therapeutic armamentarium majorly focuses on facial acne as there is a paucity of data on the management of truncal acne [15]. Prioritization of facial acne treatment, reluctance to reveal truncal areas during consultations, limited consultation time, and inadequate inquiry from dermatologists about areas affected by acne are the challenges encountered in identifying truncal acne [16]. Trifarotene is the newest retinoid introduced 20 years after the introduction of the last retinoid for acne management. It has received approval for use in the United States, Canada, United Kingdom, India, and several European countries including France, Germany, Italy, Belgium, Spain, Portugal, Sweden, the Netherlands, Finland, Norway, Denmark, Austria, Greece, Ireland, and Switzerland [17-20] and has selective action on the retinoic acid receptor (RAR)-γ, lower activity on RAR-α and RAR-β, and no activity on retinoid X receptors (RXRs). This leads to reduced expression of the genes that are involved in keratinization, immune modulation, retinoid metabolism, apoptosis, and epidermal proliferation [21,22]. The drug has comedolytic and anti-inflammatory action in humans and was also demonstrated to have depigmentation action in an in vivo study on mice [21-23]. Literature provides evidence on the safety, effectiveness, and tolerability of trifarotene in moderate-to-severe acne management with a special focus on facial and truncal acne [21].

This document aims to provide insights from an Indian Dermatology Experts' Meeting on challenges faced in the management of facial and truncal acne and the role and place in therapy of trifarotene in the management of acne in Indian patients.

## Review

An expert panel pre-meeting and an advisory board meeting were conducted to get insights from the experts regarding the burden of acne, unmet needs of acne, acne management, and the role and place of trifarotene in therapy in the management of acne in Indian patients.

Pre-meeting

An extensive search was conducted in the electronic database to explore the existing evidence on the burden of acne, unmet needs of acne, current management of facial and truncal acne, and the role and place of trifarotene in therapy in the management of acne in Indian patients. A set of pre-meeting questionnaires based on the literature search was framed and the meeting invite was extended to the nine panelists who were dermatologists by qualification. All panelists were registered on a virtual platform. The pre-meeting responses obtained are presented in Table 1.

**Table 1:** Pre-meeting questionnaire responses from panelists on the six domains. QoL: quality of life

Domain	Key points based on the response
Burden of acne	Out of the total acne patients presenting to the experts’ clinic, the overall presentation of facial acne, truncal acne, and facial along with truncal together was 20–90%, 5–10%, and 15–60% respectively. Most practitioners routinely screen for truncal acne in their patients.
Impact on QoL	Acne scarring has a negative impact on patients’ QoL [11]. It affects their self-esteem and professional opportunities and causes distress and discrimination in the workplace [11].
Unmet needs	Treatment: Limited treatment options available for truncal acne; No definite period for treatment effectiveness [24,25,26,27]. Complications: Management of acne recurrence [28]; Adverse effects associated with long-term treatment. Patient-related [16,29]; Lack of patient awareness; High cost of treatment; Poor adherence to treatment; Difficult application procedure of topical medications (especially for the truncal area); Need for an applicator device.
Place of trifarotene in the management of acne	Monotherapy in grades 1 and 2 acne patients [30]; In combination with oral medications in grade 3 and grade 4 acne patients [31]; Patients not responding to adapalene and tretinoin [23]; Patients with inflammatory facial and truncal acne [30,32]; Patients with truncal acne [33]; Maintenance regimen after recovery.
Ancillary care	Cleansers, moisturizers, and sunscreens are crucial for managing acne and treatment complications (dryness and skin irritation [30,31,32,34,35].

The pre-meeting responses provided an insight into the burden of acne and its impact on patients’ QoL, the unmet needs among patients related to treatment and associated complications, the place of trifarotene in therapy and the challenges that can be encountered in Indian patients, and the role of ancillary care in acne management.

These responses were used to construct a discussion guide for the advisory board meeting.

Advisory board meeting

The advisory board comprised nine experienced dermatologists from across India. The meeting agenda was to discuss three topics: (i) the burden and unmet needs of acne management, (ii) current management practices and the place of trifarotene in therapy in Indian patients, and (iii) personalized acne treatment and management. Each topic was discussed by the respective speakers followed by a panel discussion. The discussion guide for the advisory board meeting is presented in Table 2.

**Table 2:** Discussion guide for the advisory board meeting

Topics	Subtopics
Burden and unmet needs of acne management	Guidelines in India for acne management; Consistency in following acne management guidelines in India; Value of acne management guidelines; Need for inclusion of truncal acne management in guidelines
Current management practices and the role and place of trifarotene in therapy in Indian patients	Approach toward the treatment of mild, moderate, and severe facial and truncal acne. Identification of patients with a scarring tendency; Prevention of acne sequelae and management of patients with scarring and PIH; Choice of oral antibiotics in treating inflammatory acne lesions Key differentiating features of trifarotene compared to other treatment options with respect to safety, effectiveness, and tolerability; Prevention of retinoid dermatitis; Ease of medication application for the truncal area; Possible contact therapy with trifarotene and preferred duration of treatment; Preferred long-term treatment duration with trifarotene; Role of trifarotene as a monotherapy; Role of combination therapy of trifarotene with another retinoid; Ancillary treatments and choice of products for acne management and treatment complications; Challenges with trifarotene in an Indian setting
Personalized acne treatment and management	Importance of personalized and prolonged acne management; Key points to be considered in personalized and prolonged acne management

The results of this expert opinion paper provide a comprehensive overview of current acne management practices, highlighting the preferred treatment modalities and strategies endorsed by leading dermatologists. The opinions also highlight the position of trifarotene in Indian practice. Tables 3, 4 highlight the opinions of experts on unmet needs and current management practices for acne among Indian dermatologists.

**Table 3:** Expert opinions on the burden and unmet needs of acne management

Sr. No.	Expert opinions
1	Acne has a high disease burden. Around 20–25% of patients in their clinics present with acne.
2	Acne is a cause of concern for both professionals and patients. Patients feel upset, embarrassed, uncomfortable, and disturbed due to acne and its sequelae, which impact their psychosocial life [11].
3	Scarring and pigmentation associated with acne have a significant impact on the QoL of patients [11].
4	Various challenges are associated with the management of facial and truncal acne [16].
5	The acne management armamentarium is limited to orally administered retinoids, antibiotics, benzoyl peroxide, dapsone, and hormonal therapies [2].
6	The guidelines are valuable and essentially important, especially for newly practicing professionals. The current guidelines need to have periodic updates every two to three years. Further, there is a need to incorporate guidelines for the management of truncal acne, both personalized and long-term management. Indian dermatologists can team up for this process [24–27].

**Table 4:** Expert opinions on the current management options for acne and role and place of trifarotene therapy in Indian patients

Sr. No.	Expert opinions
Current management options for acne	
1	Patients should always be asked about the presence of truncal acne.
2	According to dermatologists, the first line of drugs for the management of grade 1 acne with mild scarring and PIH (Post-inflammatory hyperpigmentation) were BPO (Benzoyl peroxide) and adapalene [24,36].
3	In the presence of a single pustule, the dermatologists preferred topical adapalene + BPO + topical minocycline.
4	For severe facial and truncal acne with inflammatory lesions, doxycycline was the preferred drug of choice at a dose of 100 mg/day for 4–6 weeks. For poor responders and recurrences, isotretinoin therapy was suggested after discontinuing doxycycline [36].
5	In severe cases of acne, an alternative approach that the dermatologists agreed on was reducing the bacterial load as the first step in acne management with a combination therapy of amoxicillin and clavulanic acid for 7–10 days [37].
6	For moderate acne, the adapalene + BPO combination was preferred [38].
7	For women, oral contraceptives were considered a treatment option. However, dermatologists stressed that patients must be screened for contraindications and informed about the side effects of oral contraceptive pills before prescribing them [25].
8	Treatment algorithms should be followed for acne management, as they provide step-by–step processes for acne management. Further, they also help in preventing medicolegal issues that may arise during the treatment.
Current management options for scarring and pigmentation	
1	Having a parent with a history of acne scars can be considered a risk factor for the development of acne scars in young patients [39].
2	Ice pick scars develop in young patients who tend to have macro comedones, which are indicators of acne scarring [40].
3	Early and effective treatment, with continuation of treatment even after acne resolves clinically, can significantly reduce the development of scars [41].
4	Evidence exists on the effectiveness of the combination of adapalene and BPO in reducing acne scars [38].
5	It may be beneficial to start isotretinoin at an early stage at a lower dose in patients with a scarring tendency to reduce the severity of acne scars [42].
6	The habit of pricking at acne and undergoing salon facial treatments should be assessed, and patients should be educated on the fallacies of undergoing such treatments [43].
7	The use of adapalene and the application of azelaic acid cream on broken lesions to reduce acne pigmentation are preferred [44].

Role and Place of Trifarotene Therapy in Indian Patients

The experts opined that, based on the available literature, trifarotene can be used to treat facial acne as well as truncal acne considering its anti-inflammatory and anti-comedogenic properties, effect on reducing sebum production, and ability to prevent early scarring and pigmentation [30,32]. For patients with active facial lesions with PIH and scars, trifarotene can be used as the first choice of treatment [34,35]. All dermatologists supported using trifarotene as monotherapy or with oral antibiotics. The combination of two retinoids of different generations (isotretinoin+trifarotene) was not suggested by any of the dermatologists during the discussion [30,31].

For managing mild-to-moderate acne, experts suggested trifarotene as monotherapy; however, for moderate-to-severe acne management, a combination therapy of trifarotene with an antibiotic (e.g. oral doxycycline 120 mg) was supported, which is also in line with the literature [30,31]. For combination therapy, doxycycline was recommended as the drug of choice. However, for patients who are intolerant to doxycycline, lymecycline can be prescribed. The experts opined that trifarotene can be used for long-term treatment as supported by the evidence of a 52-week follow‑up trial [32]. Effective acne management with trifarotene involves educating patients on the regular use of a gentle cleanser, moisturizer, and sunscreen to enhance tolerability [30-32,34,35]. The duration of trifarotene use can vary depending on the severity of acne and other related factors and is ultimately left to the dermatologist's discretion. However, according to the literature, the duration of application mentioned in the majority of studies is up to 12 weeks [30-32].

The challenges in acne management and the role of ancillary products in preventing retinoid dermatitis are highlighted in Table 5.

**Table 5:** Challenges in the acne management and role of ancillary products

Sr. No.	Expert opinions
Challenges in acne management	
1	Application challenges, particularly in the truncal area exist [16].
2	Partners, caregivers, and family members can help with the application of the cream to the truncal area.
3	The vehicle of trifarotene cream allows for easy spreadability, making it suitable for truncal areas. The vehicle contains Simulgel® 600 polyhydroxy acid (PHA), which is nongreasy and allows for a homogenous spread and dispersion of the active ingredients of the cream. The emollients, cyclomethicone and triglycerides, provide greater spreadability, and allantoin acts as a protectant and a skin conditioning agent [45].
Role of ancillary products in preventing retinoid dermatitis	
1	The use of ancillary products is an essential part of acne treatment [30].
2	The use of a gentle cleanser, moisturizer, and sunscreen, along with a gel-based moisturizer during the summer and a cream-based moisturizer in the winter is recommended for the prevention of retinoid dermatitis [30-32,34,35].

Personalized Acne Management and Treatment

Regarding the opinions on the importance of personalized acne management, dermatologists opined that interaction with the patients is essential, and there should be a good patient-doctor relationship focusing on adequate patient counseling [46]. Further, an understanding of the psychological impact of lesions on the face and/or trunk, scarring of acne, hyperpigmentation, and erythema was emphasized as being necessary [46].

The experts opined that there are shortcomings in the guidelines as they do not consider certain patient characteristics such as comorbidities, seborrhea, duration of acne, history of previous acne, exercise routine of the patients, and supplement usage by the patients. There is no consideration for transgender patients and newly diagnosed patients versus previously treated patients [46,47]. The dermatologists expressed their views on personalized acne management approaches and mentioned that it should comprise assessing (assessing the extent of the impact of acne on a patient’s life), establishing (establishing the patient’s perspective on issues bothering them due to acne), and planning (planning treatment goals, motivating patients for adherence and long-term treatment) [46]. Emphasis was also made on Personalized Acne Treatment Tool (PATT), a novel tool developed for facilitating personalized management of acne based on patient characteristics, such as acne sequelae, location of acne, and its severity [47].

Discussion

Burden and Unmet Needs of Acne Management

Acne is one of the most prevalent inflammatory skin conditions across the world [47]. A study conducted in the Indian population reported acne in 73% of children aged 11-19 years with mild acne in 82%, moderate in 17%, and severe form of acne in 1% of the children [48]. The condition presents a peak incidence from ages 16-19 years in boys and 14-17 years in girls [49]. The increased reported incidence of acne in this age may be because this group has improved access to knowledge about the condition and/or treatment options and is more likely to seek specialized medical care [48]. Nevertheless, a few adults may continue experiencing acne in their 30s-50s [50].

**Figure 1:**
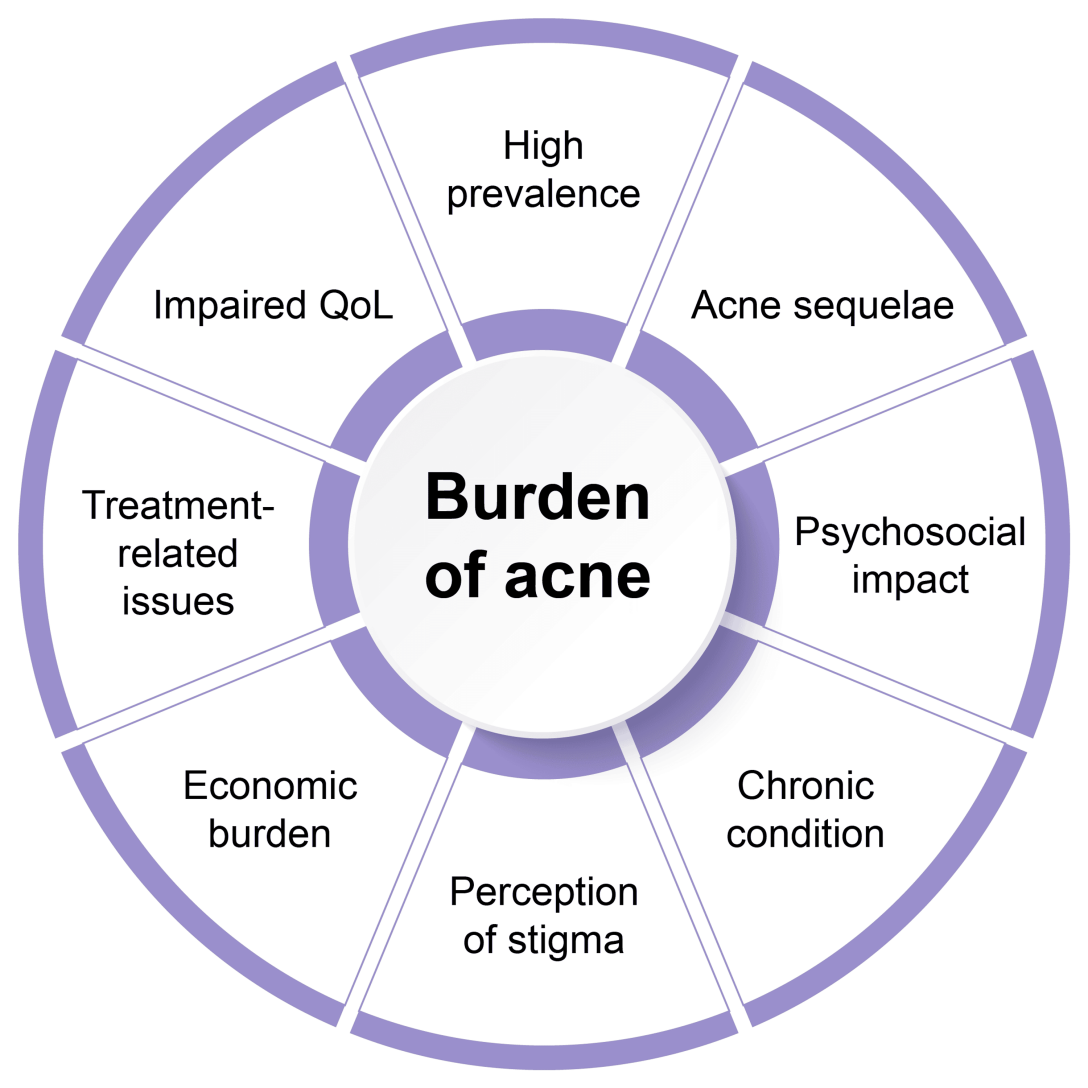
Factors responsible for the burden of acne QoL: quality of life References: [47,51,52] Image Credit: Authors

Along with the high prevalence of the condition, factors such as clinical sequelae of acne, its psychosocial impact and chronic nature, social stigma, economic burden due to direct and indirect costs, antibiotic resistance due to long-term treatment, and treatment-related adverse effects also contribute to the burden of acne (Figure 1) [47]. Patients are often reluctant to report truncal acne and reveal their body parts during clinical examination, thereby leading to underdiagnosis of truncal acne [16]. The condition has a detrimental effect on patients’ QoL [51]. A study reported a Dermatology Life Quality Index (DLQI) score of 5.97 (range: 1-12), which indicated a small to very large effect on patients’ QoL and presented an increase in score with an increase in acne severity [52]. The impact was higher among patients with facial and truncal acne compared to those with facial acne alone [53].

Various guidelines have been put forward for the management of facial acne but there are only a few guidelines on truncal acne management. The American Academy of Dermatology (AAD) recommends the topical application of a thin film of a combination of adapalene and BPO once a day after washing the area affected by acne. However, there are gaps in these AAD guidelines with respect to the management of sequelae of acne and the treatment of people with differences in skin color, which is common in Indian populations [24,54]. The Global Alliance International Group recommends oral antibiotics for inflammatory acne involving truncal areas that fail to respond adequately to typical therapy [55]. A combination of systemic therapy and topical therapy is recommended by Canadian practice guidelines for truncal acne [25]. Although these guidelines discuss the management of truncal acne, the data are inadequate as the guidelines only report on the management of areas amenable to topical therapy but do not consider the severity of the acne or patient characteristics. Further, Indian guidelines for acne management, which were published in 2009, have not been updated yet [27], thereby stressing the need for periodic updates of the guidelines.

The varied presentations of acne and its severity have a negative impact on patients’ QoL. Despite the differences in terms of patient characteristics, their expectations of the treatment, and the effectiveness of the drug, the current guidelines do not provide a personalized plan and mainly focus on treatment based on acne severity (mild, moderate, or severe) [24,46]. In a qualitative study, patients expressed their frustration and reported that they felt pressured to follow a treatment plan they were not comfortable with. Good patient-healthcare professional (HCP) interactions and understanding of patients’ concerns are important factors for the success of treatment [56].

Acne is a chronic condition that has the characteristics of a prolonged course, relapse and recurrence patterns, slow onset, acute outbreak, and psychosocial impact on QoL as defined by the World Health Organization (WHO). Thus, the condition requires a long course of treatment, which can range from months to years [57]. Additionally, scarring and post-inflammatory hyperpigmentation are common complications of acne that affect most patients and vary in severity based on acne grading [40,58]. Post-inflammatory hyperpigmentation is the overproduction of melanin or abnormal melanin deposition in the dermis or epidermis following inflammation, resulting in blue-gray discoloration of the skin [59]. These complications are of concern even more than the condition itself as their appearance causes psychological stress, embarrassment, low self-esteem, feelings of unattractiveness, increased anxiety and anger, and depression in patients. Despite its emotional and psychological burden, permanent disfigurement, and about 95% of patients experiencing complications, there is no universal solution available for acne sequelae [58,60].

Current Management Options for Acne

The current armamentarium of acne treatment consists of topical and systemic therapy. Topical treatment comprises BPO, antibiotics (clindamycin and erythromycin), dapsone, azelaic acid, and retinoids (adapalene, tazarotene, and tretinoin), while systemic treatment comprises the use of systemic antibiotics (penicillin, macrolides, trimethoprim, cephalosporins, tetracyclines, and sulfamethoxazole), isotretinoin, and hormonal agents (antiandrogens, combined oral contraceptives, and corticosteroids) [61]. The AAD recommends BPO alone or in combination with antibiotics for the management of mild acne, and in combination with topical retinoids or systemic antibiotics for the management of moderate-to-severe acne [24]. The European guideline categorizes patients with comedonal acne, mild-moderate papulopustular acne, severe papulopustular/moderate nodular acne, and severe nodular/conglobate acne, and recommends the use of topical retinoids for comedonal acne, a combination of adapalene/clindamycin with BPO for mild-moderate papulopustular acne, and isotretinoin for both severe papulopustular/moderate nodular and severe nodular/conglobate acne. The guideline mentions that the truncal area plays an important role in therapeutic decision-making [38].

Per Canadian guidelines, comedonal and mild papulopustular acne should be managed with BPO/topical retinoids or both: a combination of BPO/clindamycin or BPO/adapalene. In the case of moderate papulopustular acne, oral contraceptives/systemic antibiotics should be added to the mild regimen. Severe nodular/papulopustular acne is managed by isotretinoin. There is an absence of information on the management of truncal acne (there is no information on truncal treatment efficacy) [25]. The guidelines of the Japanese Dermatological Association emphasize fixed-dose gel combination therapies with BPO for acute inflammatory lesions. Monotherapy with antibiotics is not preferred, and the maintenance phase comprises a combination of 2.5% BPO and 0.1% adapalene [26].

Similarly, monotherapy with topical antibiotics is discouraged by the Dermatological Society of Singapore, and combination therapy of topical adapalene + BPO is preferred over monotherapy with either of the drugs for facial acne. Topical retinoids for inflammatory and comedonal acne are the first line of therapy. Retinoids are also recommended as the drug of choice in the maintenance phase of acne. Systemic antibiotics such as doxycycline, erythromycin, and tetracycline, with topical agents are indicated in cases of moderate-to-severe acne, while isotretinoin is indicated in the management of severe acne [36]. Evidence from a network meta-analysis reported the use of topical treatment combinations, photochemical therapy, and chemical peels to be the most effective treatment for patients with mild-to-moderate acne, while a combination of topical drugs and oral antibiotics as well as photodynamic therapy with oral isotretinoin is reported to be effective against moderate-to-severe acne [62].

Place of Trifarotene Therapy in Acne Management in an Indian Setting

Trifarotene (0.005%), a topical retinoid, is a fourth-generation FDA‑approved novel drug introduced in the acne management armamentarium after 20 years of third-generation tazarotene drug approval [63]. With its strong selectivity for RAR-γ, low activity on RAR-α (65-fold lower) and RAR-β (16-fold lower), and no activity on RXRs, trifarotene downregulates the acne gene expressions and exerts its comedolytic, depigmentation, and anti-inflammatory actions. It influences three pathways-skin hydration influencing the skin barrier, cell adhesion causing comedolytic action, and proteolysis downregulating metalloproteinases and improving the texture of the skin [21]. Of all the properties reported in the literature, trifarotene has superior anti-pigmentation activity on the pigment induced by ultraviolet radiation compared with adapalene as demonstrated in an in vivo study using a mouse tail system [23]. The main indication for the drug is in the presence of facial and/or truncal acne and patients requiring a rapid response from the treatment [63].

The plasma half-life of trifarotene is five minutes, and the drug undergoes quick hepatic metabolism by microsomal enzymes compared with adapalene, which has a half-life of less than one hour [28,30]. The minimized blood concentration of trifarotene allows its use on large body surface areas such as the trunk, that require the diffused application of the drug [64]. Further, the trifarotene vehicle cream has easy spreadability, making its texture suitable for truncal application [65].

Therapeutic studies by Del Rosso et al. [31], Dreno et al. [66], Blume-Peytavi et al. [32], Tan et al. [30], Wagner et al. [33], Schleicher et al. [34], and Alexis et al. [35] have confirmed the safety, effectiveness, and tolerability of trifarotene in the management of facial and truncal acne in both adult and pediatric populations. The adjuvant skincare products that were used by the patients in the studies are presented in Table 6.

**Table 6:** Studies reporting the safety, effectiveness, tolerability of trifarotene, and use of adjuvant skincare (CTMP) products in the management of facial and truncal acne CTMP: cleansing, toning, moisturizing, and protecting; PIH: post-inflammatory hyperpigmentation; QoL: quality of life; SPF: sun protection factor.

Study	Use of trifarotene	Use of noninvestigational products
Tan et al., 2019 [30]	50 µg/g of trifarotene once daily for 12 weeks in patients aged ≥9 years with moderate facial and truncal acne was safe (no serious adverse events), effective (reduced lesion counts and success in 29–43% of patients), and well tolerated compared with the vehicle cream.	Cleanser and moisturizer
Blume-Peytavi et al., 2019 [32]	50 µg/g of trifarotene once daily for 12 weeks (follow-up at 52 weeks) in patients aged ≥9 years with moderate facial and truncal acne was safe (1% serious adverse events), effective (success in 58% of patients), well tolerated, and improved QoL.	Non-comedogenic, hypoallergenic moisturizer
Wagner et al., 2020 [33]	Once daily 50–100 µg/g trifarotene in patients with moderate-to–severe facial and truncal acne was safe (no cardiovascular effects or drug–drug interaction in potential childbearing women), effective, and well tolerated (100 µg/g was less tolerated than 50 µg/g).	Not reported
Dreno et al., 2021 [65]	0.005% of trifarotene was used daily for four weeks by patients aged 18–35 years with moderate inflammatory back acne. Biopsies revealed that 67 genes responsible for cellular migration, inflammation, and extracellular matrix reorganization were uniquely downregulated by trifarotene, otherwise not observed with spontaneously resolving acne.	Not reported
Del Rosso et al., 2022 [31]	50 µg/g of trifarotene + 120 mg of enteric-coated doxycycline once daily for 12 weeks among patients ≥12 years of age with severe facial acne was safe (mild adverse events) and effective (reduced lesion counts and success in 32% of patients) compared with placebo and vehicle cream.	Moisturizer with SPF 30, gentle noncomedogenic cleanser, and moisturizing lotion
Schleicher S et al., 2023 [34]	50 µg/g of trifarotene once daily for 24 weeks in patients aged 17–34 years with moderate-to–severe facial acne with acne scars had a significantly greater reduction in total atrophic scar (-5.9) compared with vehicle cream (-2.7).	Oil-absorbing moisturizer with SPF 30, gentle skin cleanser, and moisturizing lotion
Alexis AF et al., 2024 [35]	50 µg/g of trifarotene once daily for 24 weeks in patients aged 13–35 years with acne-induced hyperpigmentation (AIH) showed a significant reduction in overall pigmentation disease severity (-34.4) compared with the vehicle cream (-23.6) at 12 weeks. However, the primary endpoint of the study was not met, as comparable effects were observed in reduction in overall pigmentation disease severity between the trifarotene (-45.4%) and vehicle (-44.9%) groups at 24 weeks.	Moisturizer with SPF 30, gentle skin cleanser, and moisturizing lotion

Challenges in the Management of Acne

Trifarotene, like first-, second-, and third-generation retinoids, has retinoid-associated adverse effects such as irritation, stinging, scaling, pruritis, erythema, burning sensation at the application site, dryness, and sunburn. These effects, although not serious, may hinder patient adherence to retinoids [29]. Treatment can be modified by applying the retinoid on alternate days and encouraging the use of moisturizers [67,68]. A consensus paper reports that a holistic skincare routine, such as cleansing, moisturizing, and sun protection (CMP), reduces side effects associated with topical therapy and improves treatment adherence [69].

Role of Ancillary Treatment in the Prevention of Retinoid Dermatitis

Retinoids are the mainstay of acne management; however, their use is associated with retinoid‑associated dermatitis, which may result in dryness, erythema, cutaneous irritation leading to peeling of the skin, and retinoid flares [2,28,68]. Although the condition is mild-to-moderate and superficial, lasting for one to two weeks after the initiation of retinoid treatment and subsiding thereafter [68]; many patients may discontinue treatment during this phase [29]. Insufficient adherence to the treatment leads to acne relapse and recurrence, an increased cost of treatment, and patient dissatisfaction.

Modern acne management strategies emphasize the use of ancillary products along with acne management drugs. The basic care essential for patients with acne includes controlling sebum secretion, preventing bacterial infection, and reducing abnormal keratinization [70]. It is important to note that while the effectiveness of trifarotene for acne vulgaris is well-documented, its use as a retinoid is also associated with retinoid dermatitis. However, an expert consensus panel comprising dermatologists from various countries, including India, has suggested a holistic care routine called CTMP (Cleansing, Treatment, Moisturization, and Photoprotection) [69]. This routine is recommended as an adjunct treatment to reduce the side effects of topical therapy, such as dryness, photosensitivity, and irritation, and to decrease the need for systemic treatments for acne. The moisturizers improve skin hydration and barrier function, while the sunscreen helps in limiting PIH [69,71]. The experts in the present study also opined on having CTMP as an ancillary care regime in acne patients. These products, when used daily, improve local tolerability, provide photoprotection, reduce scaling and drying, and can have a significant impact on patient satisfaction, adherence to the treatment, and QoL [28,69,71,72].

Personalized Acne Management and Treatment

Acne management customized according to individual patient requirements is essential as each patient presents with varying characteristics of the condition that have different degrees of impact on the patient’s QoL. However, the guidelines available do not provide sufficient information on personalized management and omit patient- and disease-related factors, while focusing majorly on clinical assessment of the visible facial lesions to define the severity of the condition [46,73]. Additionally, these guidelines majorly focus on facial acne with truncal acne management remaining unexplored [38,55]. Personalized care is essential for patient adherence to the treatment and achieving a successful outcome. Adherence is further influenced by factors such as patient characteristics, the relationship between the patient and their HCP, the route of administration of the drug, the frequency and duration of treatment, tolerability toward the drug, and the effectiveness or side effects experienced by the patients. Thus, the development of a treatment plan considering these factors is important [46].

An expert panel based on consensus developed a personalized acne care pathway focusing on the patient-HCP relationship, treatment selection based on patient characteristics, review and assessment of treatment outcomes, and modifying them whenever required during the follow-up [46]. Per the Personalizing Acne: Consensus of Experts (PACE) recommendation, HCPs should discuss with their patients the safety, efficacy, and tolerability of the treatment, the patient’s expectations from the treatment, the administration route and technique of drug application, the daily skincare routine to be followed, the importance of adherence to the treatment, the risk and impact of acne sequelae, access to the treatment, and the affordability of the treatment [46]. Further, the treatment selection should be based on the type and severity of acne, its location (facial or truncal), the risk of sequelae, patient preference, and antibiotic resistance. Any modifications to the treatment plan (switching the treatment, escalating the treatment, de-escalating the treatment, or stopping the treatment) should be considered based on the response to the treatment, patient satisfaction, associated adverse effects, duration of treatment, and the patient’s desire to shift to another treatment [46]. PATT is a novel tool developed for facilitating personalized management of acne based on PACE recommendations and provides a higher quality of acne management compared with the existing guidelines [73].

This study has certain limitations. It does not provide direct evidence on the safety and efficacy of trifarotene in the management strategies of acne vulgaris in clinical practice. Further, the practices reported by the dermatologists may vary significantly based on their individual clinical experiences and patient demographics. Apart from these limitations, it should be mentioned that this study reflects the opinions and practices of dermatologists at a specific point in time; however, these practices may evolve over time. Additionally, the reliance on expert judgment in this study introduces the possibility of bias, as varying viewpoints and preferences may have influenced the reported results. Therefore, it is essential to consider these limitations when evaluating the findings of this study and to conduct further research to validate and expand upon the results.

## Conclusions

Acne has a psychosocial impact on patients, may lead to an increased economic burden, and may significantly impair patients’ QoL. Based on scientific literature, the use of trifarotene as a topical agent can be promising for the management of facial and truncal acne in an Indian setting. Monotherapy can be considered for facial and truncal acne vulgaris, while combination therapy with antibiotics can be used to manage severe acne. Robust clinical data on the effectiveness, safety, and tolerability of trifarotene in the management of both facial and truncal acne and its selective action on RAR-γ receptors makes trifarotene a valuable addition to the treatment armamentarium for patients with acne in India.
